# The Soundscape Quality in Some Urban Parks in Milan, Italy

**DOI:** 10.3390/ijerph10062348

**Published:** 2013-06-06

**Authors:** Giovanni Brambilla, Veronica Gallo, Giovanni Zambon

**Affiliations:** 1 CNR Institute of Acoustics and Sensors “O.M. Corbino”, Via del Fosso del Cavaliere 100, Roma 00133, Italy; E-Mail: veronica.gallo@idasc.cnr.it; 2 Deparment of Earth and Environmental Sciences, University of Milan-Bicocca, Piazza della Scienza 1, Milan 20126, Italy; E-Mail: giovanni.zambon@unimib.it

**Keywords:** soundscape, perceived quality, urban parks

## Abstract

Urban parks play an important role in preserving and promoting the health of citizens who are often exposed to noise pollution and the stress of daily life. The present study describes the main results obtained from a survey performed in five urban parks in Milan. Measurements of the acoustic environment were carried out in 29 sites together with interviews with 231 users on certain aspects of the parks not limited to merely sound. Acoustic data show that the surveyed parks mostly do not comply with the noise limit issued by the Italian legislation on protected areas. The unweighted 1/3-octave spectrum centre of gravity G and L_A50_ perform satisfactorily in discriminating among the acoustic environments. Such clear distinction was not observed in the subjective ratings on the perceived quality of the soundscape, likely due to the influence by non-acoustic factors that act as mediators in the assessment. This hypothesis is supported by the collected data on the perceived quality of quietness, which was rated worse than that of the soundscape. Comparing acoustic data with ratings, the perceived quality of the total environment was found to be less dependent on L_Aeq_ than soundscape and quietness.

## 1. Introduction

Since the introduction of the “soundscape” concept by R.M. Schafer in the 1970s [1], many projects, e.g., the COST Action “Soundscape of European Cities and Landscapes” [2], and studies have dealt with the perception of the acoustic environment in a context, that is considering the interrelationships between person, activity, and place, in space and time. Thus, soundscape research is a step forward in noise control, as it does not conceive noise *per se* but rather reconceives the conditions and purposes of its production, perception, and evaluation, accounting for a human-centred point of view [3]. For this reason the soundscape approach treats the acoustic environment as a multi-dimensional entity composed of several audible sources, some of which enhance and others diminish the effects on overall soundscape quality [4]. In 2003, Lercher and Schulte-Fortkamp reviewed the relevance of soundscape research for the assessment of noise annoyance at the community level [5]. The soundscape approach can provide new insights into the existing annoyance data and new holistic research strategies having as a central issue the improvement of the relationship between the “aural space” and the living environment [3].

An interesting application of the soundscape approach deals with parks, particularly those in urban areas. Because Nature plays a vital role in human health and well-being, parks play an essential health-promoting role by providing individuals access to Nature [6,7]. Indeed, parks can help individuals recover, at least temporarily, from stress [8], and they offer opportunities for relaxation from the noise pollution to which the population is exposed in daily life. Unfortunately, urban parks are often surrounded by noisy areas due to the sound emission of road traffic, industries, and other sources. The extent of the park has an important influence on the tranquillity that can be achieved due to effects of distance in reducing traffic noise emanating from the boundary roads and the provision of a high percentage of natural features [9].

Awareness of the preservation and improvement of the environment quality in urban parks is increasing, as also addressed by the European Directive 2002/49/EC on the assessment and management of environmental noise [10], which, among its objectives, requires the preservation of environmental noise quality where it is good.

The studies adopting the soundscape approach in analysing the acoustic environment in urban green areas have been increasing in the last few years; for instance, see [4,11,12,13,14,15,16]. Among the several outcomes, good soundscape quality was found to be achieved if the road traffic noise exposure is below 50 dB(A) during daytime [4]. The importance of masking by natural sounds, such as sound produced by water [17,18], in improving the quality of the city park soundscape has also been reported [13]. Considering the non-acoustic factors, the reasons for coming and the activities carried out in the park have been shown to influence the perceived restoration achieved [12,19].

In Italy, there are few studies on city parks. The first investigation available in literature on the soundscape in urban parks was performed in three green areas in Naples [20]. The study found that noise levels were greatly influenced by the traffic on the surrounding busy roads, and the reported annoyance was also influenced by the subject’s expectation of hearing a sound in a specific environment.

The present study is an extension of the investigation carried out in Naples [20], as it was carried out using a similar experimental protocol, but the questionnaire used for the interviews to park users was modified and expanded on the basis of the previous experience. In particular, self-assessment of noisiness perceived at home and at workplace was added to investigate the influence of these environments on the ratings given on the other items. Measurements of the acoustic environment were performed in five parks in Milan. These parks differ from those in Naples because of the socio-cultural and climatic context and because the answer options provided for questions dealing with non-acoustic factors were different. The set of acoustic data and subjective ratings was more extensive than the investigation in Naples, and the statistical analysis was carried out in greater detail.

## 2. Method

**Figure 1:**
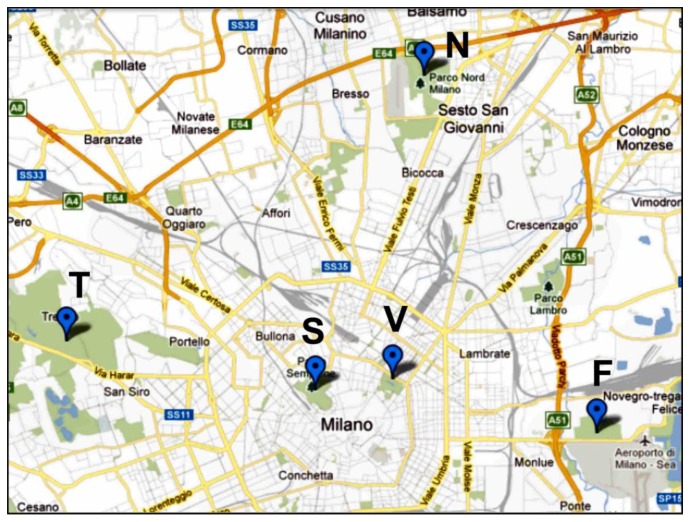
Locations of the five parks in Milan.

The five parks selected in Milan are listed in Table 1 together with the predominant sound sources present during the survey and the sound monitoring. The locations of the parks within the city are shown in Figure 1.

**Table 1:** Urban parks in Milan surveyed in the study.

Park	Code	Location	Predominant sound sources
Forlanini	F	Suburban	Road traffic and aircraft fly-overs
Nord	N	Suburban	Road traffic and aircraft fly-overs
Sempione	S	Central area	Road traffic
Trenno	T	Suburban	Road traffic and aircraft fly-overs
Venezia	V	Central area	Road traffic

Forlanini Park (F in Figure 1) is an urban garden on the outskirts of Milan close to Linate airport. This park has an area of 75 ha with no fence and is ideal for sporting activities such as jogging and biking. The “Lambro” river flows in the western part of the park and the “Salesina” pond is located in the eastern part.

Milan North Park (N in Figure 1) is a regional park of 600 ha in the northern suburbs of Milan. The park has 350 ha of green, including wooded areas, bushes, hedges and a small stretch of water. Free playgrounds are present together with several sports facilities (cycle track, Breda stadium, giant chess board, baseball, soccer, and basketball).

Sempione Park (S in Figure 1) is a historical urban park in downtown Milan. The park has an area of 38.6 ha and is completely bounded. Because of its location, it plays an important role in arts and free time for the citizens of Milan. Due to its proximity to the “Sforzesco” castle, the park is also a tourist attraction. The park area includes the Palace of Arts, the Civic Arena, the Milan Aquarium and the White Tower.

Trenno Park (T in Figure 1) has an area of 59 ha and is located in the western zone of Milan near the hippodrome. This park is a small part of a wider protected natural area (46,300 ha). Due to its flat surface, the park is ideal for training in athletics and biking. The park is crossed by an asphalt road beside a traditional fountain. Inside, there are facilities such as a botanical trail, playgrounds and bars.

Porta Venezia Park (V in Figure 1), currently named the Indro Montanelli (a famous Italian writer) Public Garden, is a historical park of 17.2 ha in the central area of Milan. It was the first park dedicated to collective entertainment and is completely bounded. Inside the park there are botanical trails, three playgrounds and two areas where dogs are allowed. Many sports can be practiced in the park, and it often hosts exhibits and public events. The Milan Planetarium and the Milan Natural Science Museum are inside the park.

### Field Survey

In each of the five parks, locations most frequented by visitors were selected to measure the acoustic environment. The measurements were carried out during daylight hours on weekdays and Saturdays, as reported in Table 2. All the measurements were taken in summer (June and July) during sunny weather with light or no wind. In the chosen periods, the parks were largely frequented due to the hot climate and because of school holidays. In the Forlanini and Trenno parks, located in suburban zones and away from residential areas, only two sites were selected for acoustic measurements because the areas frequented by visitors were small and not largely attended. This was the reason for the limited number of interviewees (see Table 3).

**Table 2:** Sites selected for the acoustic measurements in the urban parks.

Park	Code	No. of sites		
		Weekday	Saturday	Total
Forlanini	F	2	-	2
Nord	N	6	3	9
Sempione	S	6	4	10
Trenno	T	2	-	2
Venezia	V	6	-	6
Total		22	7	29

**Table 3:** Subjects interviewed in the urban parks.

Park	Code	No. of subjects		
		Weekday	Saturday	Total
Forlanini	F	15	-	15
Nord	N	33	58	91
Sempione	S	44	30	74
Trenno	T	14	-	14
Venezia	V	37	-	37
Total		143	88	231

To match as closely as possible the subjective responses of park users to their experienced sound exposure in the park, face-to-face interviews were carried out with those present around the site where the acoustic monitoring was in progress simultaneously with the sound measurement. For this reason, the acoustic monitoring at each site lasted approximately 30 min, during which the operator took note of the predominant sound sources and of those producing noise events clearly audible in the ambient noise. Different sound sources were noticed during the noise measurements, including non-natural sources such as road traffic noise and aircraft fly-over.

The acoustic data collected included the time history of 1 s L_Aeq_, the corresponding 1/3-octave band spectrum, the equivalent sound level L_Aeq_ in reference to the measurement time, some statistical levels (L_A10_, L_A50_, L_A90_, L_A95_), the L_A10_-L_A90_ difference as a descriptor of the “acoustic climate”, the number of sound events exceeding by 3 dB the statistical level L_A50_ (an index that correlates well with the number of vehicles heard at close distance [21]), and the unweighted spectrum centre of gravity G, proposed as a good measure for the degree of pollution of the acoustic environment with traffic noise [20] and calculated according to the following relationship [22]:

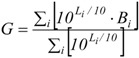
(1)
where *L_i_* is the unweighted sound pressure level in dB, measured for each 1/3-octave band-width *B_i_* from 80 up to 8,000 Hz, as this frequency range includes most of the urban sonic environments.

The interviewees were selected randomly, and data were collected from 231 respondents, as reported in Table 3. The characteristics of the interviewees are summarised in Table 4, where the percentage in reference to the overall sample is also given.

**Table 4:** Characteristics of the respondents in the urban parks and percentage in reference to the total sample.

Park	Code	Age			Gender	
		18–30	30–60	>60	Male	Female
Forlanini	F	2	5	8	8	7
Nord	N	47	32	12	54	37
Sempione	S	35	28	11	47	27
Trenno	T	5	6	3	8	6
Venezia	V	9	17	11	20	17
Total		98	88	45	137	94
		(42.4%)	(38.1%)	(19.5%)	(59.3%)	(40.7%)

The interviews were carried out according to a questionnaire modified from that applied in the above-mentioned study in Naples [20]. The questionnaire consisted of sixteen questions covering the following items:

- interviewee’s personal data (age, gender, occupational status);
- presence in the park: (i) preferred days to visit the the park (weekdays or weekends), (ii) weekly frequency (once, twice, all days, or occasionally), (iii) average duration of visit (less than one hour, between one and two hours, or more than two hours);
- main reason for frequenting the park selected among seven answer options (reading, children, pets, walking, sport, nature, or tranquillity);
- other urban parks frequented;
- importance of certain aspects of a park that affect its pleasantness, namely, vegetation, clean air, cleanliness, security and quietness (explained to interviewees as the absence of “disturbing” sounds [23]);
- rating the quality of the above aspects for each surveyed park, given on a scale from 1 (bad) to 5 (very good);
- sounds expected to be heard in the park (voices, road traffic, aircraft fly-overs, or dogs barking) and annoyance due to these sounds given on a scale from 1 (not at all annoyed) to 5 (very much annoyed);
- perceived quality of the area as a whole (explained to interviewees as the total environment) and of its soundscape (explained to interviewees as the perception of the acoustic environment), given on a 5-point scale from 1 (bad) to 5 (very good);
- assessment of noisiness at home and at work, given on a 5-point scale from 1 (not at all) to 5 (very much).

## 3. Results and Discussion

### 3.1. Acoustic Environment

**Figure 2:**
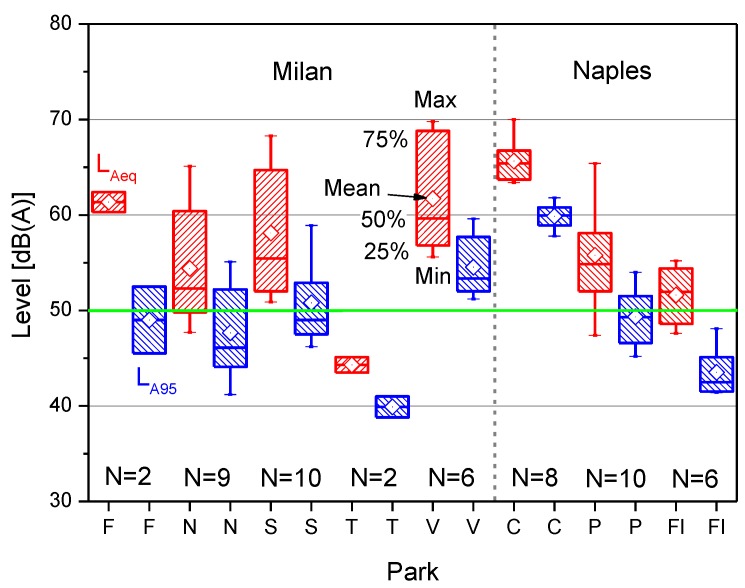
Box plot of L_Aeq_ (red) and L_A95_ (blue) levels measured in the parks in Milancompared with values observed in Naples [20]. (N = number of sites, green line = Italian daytime L_Aeq_ limit).

The variability of L_Aeq_ values measured in the selected sites chosen in the five parks is shown in Figure 2, where the box plot also reports the distribution of L_A95_ together with the number of measurement sites N for each park. The highest levels are observed for Venezia Park (V), located downtown and surrounded by busy roads, whereas Trenno Park (T) shows the lowest levels, likely due to its suburban location. It should be noted that, according to the Italian legislation on acoustic zoning [24], parks are classified as protected areas and that the daytime L_Aeq_ (6–22 h) must not exceed the limit of 50 dB(A). Figure 2, where this limit is represented by the green line, shows that the limit was exceeded at most of the surveyed sites. For instance, in Venezia Park the L_Aeq_ range is 56–70 dB(A) and the L_A95_ is never below 50 dB(A). Excluding the two parks where few measurements were taken (Forlanini and Trenno), the standard deviations of L_Aeq_ values were similar (approximately 6 dB). In addition, the distributions of the L_Aeq_ values show a slightly positive skewness (0.4–0.7), that is, the bulk of the values lie to the left of the mean. For comparison Figure 2 also shows the L_Aeq_ and L_A95_ variability observed in the previous study carried out in the three parks in Naples [20]. The values in these parks are within the range of those measured in the parks of Milan.

**Figure 3:**
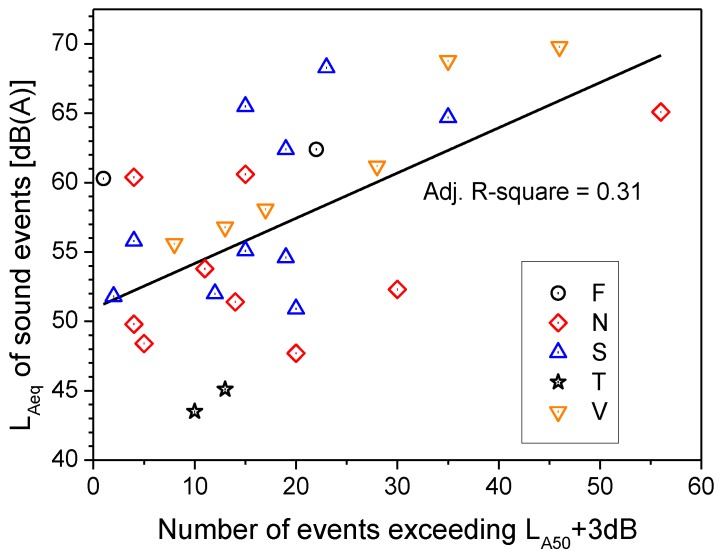
L_Aeq_ and number of sound events observed in the parks.

Because several sound events, including those produced by non-natural sources, were recorded in the ambient noise, the resulting soundscape may have been perceived to lose its feeling of quietness. These events were detected by the exceedance of the value L_A50_ + 3 dB, a threshold well correlated with the number of sound events produced by vehicles heard at close distance [21]. Indeed, most of the surveyed parks were surrounded by busy roads. The number of such events is plotted *versus* L_Aeq_ in Figure 3, which also shows the linear regression of data and the adjusted R^2^. The L_Aeq_ values increase with the number of events at a rate of 3 dB for every increment of approximately 10 events.

To look for common features of the acoustic environments of the parks, the sound descriptors L_Aeq_, L_A10_, L_A50_, L_A90_, L_A95_, the difference L_A10_-L_A90,_ and the unweighted spectrum centre of gravity G determined for the 29 sites were used as inputs for the hierarchical cluster analysis. This analysis was performed using the SPSS software on the above data normalised by the following algorithm:

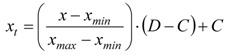
(2)
where *x_t_* is the normalised value with a range between *C* and *D* (set to C = 0 and D = 1) of the input variable *x*, which is between the maximum and minimum values *x_max_* and *x_min_*. The Ward algorithm for agglomerative clustering was applied; at each step, the method merges the pairs of clusters with minimum cluster distance. Because the Ward method was selected, the squared Euclidean distance was set as a metric of distance between pairs of observations. The range of solutions for clustering was chosen between eight and two groups, and that corresponding to five clusters was chosen for a straightforward comparison with the belonging of the sites to each park. Analysis of variance (ANOVA) showed that all seven input variables were significant for the above clustering. To check the robustness of the clustering output, the K-means procedure was also applied, taking as initial centroids those resulting from the hierarchical clustering and the same number of clusters. The results were identical to those previously obtained, confirming the robustness of the chosen clustering. As shown in Table 5, there is no complete correspondence between the sites in each park and their cluster membership. The majority of sites in Venezia Park are in cluster 1, whereas all the sites in Trenno Park and the majority of those in Nord Park are in cluster 3, whose groups had the highest number of sites.

**Table 5:** Percentage of sites in parks occurring in each cluster.

Park	Cluster membership				
	1	2	3	4	5
	(8 sites)	(2 sites)	(12 sites)	(5 sites)	(2 sites)
F	50.0	50.0	0	0	0
N	22.2	0	66.7	11.1	0
S	10.0	10.0	40.0	20.0	20.0
T	0	0	100	0	0
V	66.7	0	0	33.3	0

**Figure 4:**
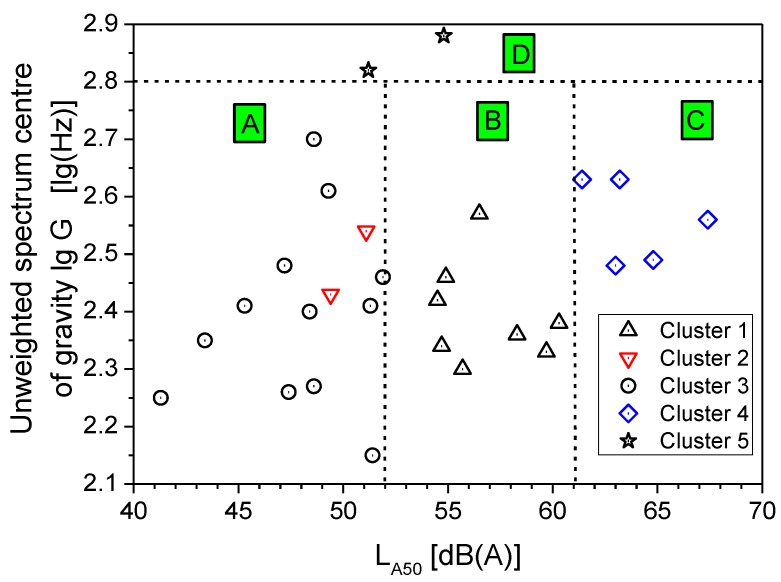
Centre of gravity G *versus* L_A50_ and cluster memberships of sites.

Among the sound descriptors used for clustering, the statistical level L_A50_ has been indicated as highly important for assessing the quality of a quiet rural soundscape [21]. The L_A50_ values measured in the parks are plotted in Figure 4* versus* the unweighted 1/3-octave spectrum centre of gravity lg(G) for all the sites. Considering the cluster memberships of the sites, a distinction can be made according to the intervals for the lg(G) and L_A50_ values. Four regions in particular, named A to D in the plot, can be distinguished according to the limit values reported in Table 6, where the percentage of sites in each cluster is given for every region. The values of lg(G) > 2.8, already proposed as indicators of good quality for a quiet rural soundscape [21], correspond in this study to the sites in Sempione Park, which were far from the border and the surrounding roads. In addition, the measurements were taken on Saturday, when the road traffic is usually less busy than that on weekdays.

**Table 6:** Intervals of the centre of gravity G and L_A50_ and the percentage of cluster memberships.

Region		Cluster membership				
		1	2	3	4	5
		(8 sites)	(2 sites)	(12 sites)	(5 sites)	(2 sites)
A	lgG ≤ 2.8 and L_A50_ ≤ 52	0	100	100	0	0
B	lgG ≤ 2.8 and 52 < L_A50_ ≤ 61	100	0	0	0	0
C	lgG ≤ 2.8 and L_A50_ > 61	0	0	0	100	0
D	lgG > 2.8	0	0	0	0	100

### 3.2. Subjective Responses

The data from the questionnaires collected in all five parks show that 61% of the interviewees reported a preference for staying in the park on weekdays and 73% frequented the park weekly (from once per week up to all days) for a visit longer than 2 h (53% of subjects). Considering these responses, together with the observed habit to visit the same park most frequently (63% of subjects), it is likely that the collected subjective ratings were outcomes of their consolidated experience of the park rather than of occasional experiences.

The most frequent reasons for visiting the park were, in descending order, seeking tranquillity (29%), walking (15%), kids and nature (14%), reading (12%), sport (9%) and pets (7%), as shown in Table 7, where the data are reported for each park and are differentiated between weekdays and Saturdays for the Nord and Sempione parks. With the sole exception of Forlanini Park, where the main reason for being in the park was largely pets, tranquillity was the predominant motivation, although this motivation was equal to that of sport at Trenno Park. Differences in motivations were observed between weekdays and Saturdays for the two parks monitored on these days (Nord and Sempione). In Nord, the motivation “tranquillity” was reported by 43% of the respondents on Saturdays and by 20% on weekdays, whereas in Sempione the corresponding values were 33% (Saturdays) and 20% (weekdays).

**Figure 5:**
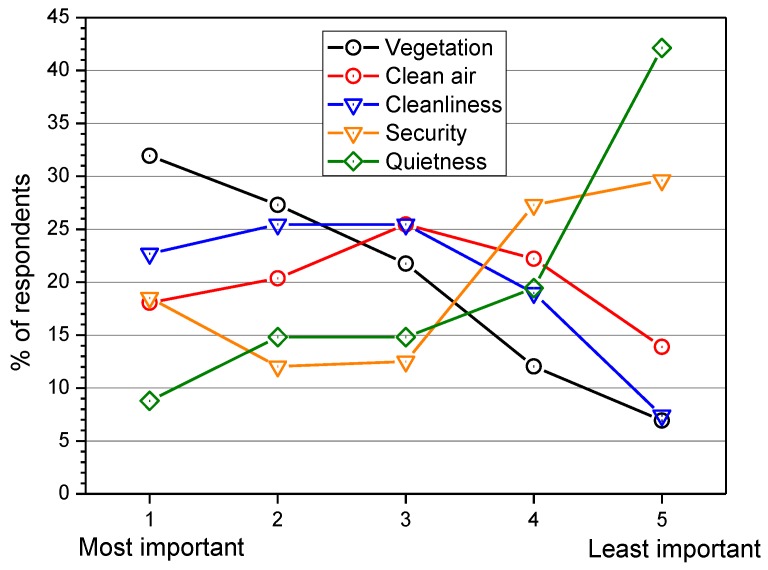
Importance of certain aspects for a park’s pleasantness.

For the pleasantness of a park, the aspects considered most and least important are reported in Figure 5. As shown, 32% of the respondents reported vegetation as the most important aspect, whereas quietness was rated the least important by 42% of the subjects. For respondents going to the park for tranquillity, quietness was rated the most and least important aspect by 8% and 39% respectively. From this outcome, the feeling of tranquillity appears to be influenced not only by quietness, but also by other non-acoustic features, such as the visual feature [25].

**Table 7:** Occurrence of main motivation for being in the park.

Park		Motivation to be in the park						
		Reading	Children	Pets	Walking	Sport	Nature	Tranquillity
F		1	1	9	3	0	1	0
N	Weekday	2	6	1	6	5	7	6
	Saturday	1	4	1	7	7	13	25
	Total	3	10	2	13	12	20	31
S	Weekday	9	8	4	8	3	3	9
	Saturday	6	3	1	4	2	4	10
	Total	15	11	5	12	5	7	19
T		1	2	0	1	4	2	4
V		8	8	0	6	1	2	12
Total		28	32	16	35	22	32	66

**Figure 6:**
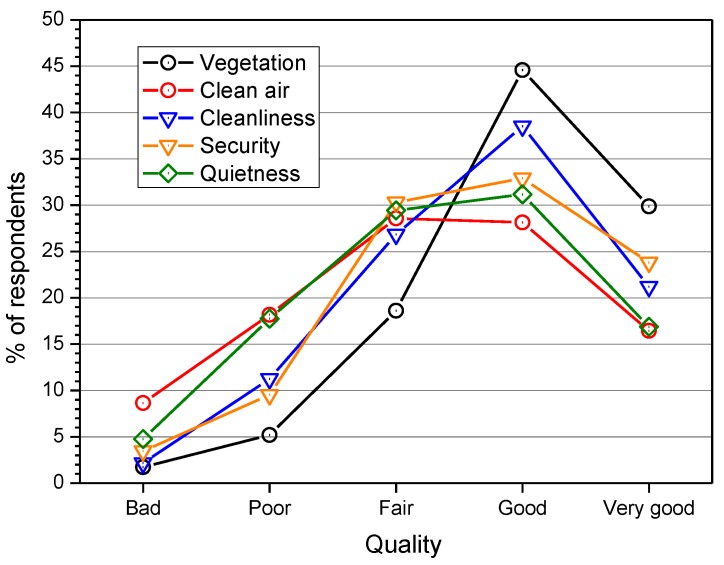
Perceived quality of certain aspects of the parks.

As shown in Figure 6, the quality of the above five aspects in the surveyed parks was largely judged positively by the interviewees. For instance, vegetation and quietness were rated “good” by 45% and 31% of subjects, respectively. Considering the 19 respondents (8% of the total sample, see Figure 5) who rated quietness as the most important aspect of a park for its pleasantness, 58% judged the perceived quality of this aspect as “good” (26%) and “very good” (32%) in the surveyed park.

**Figure 7:**
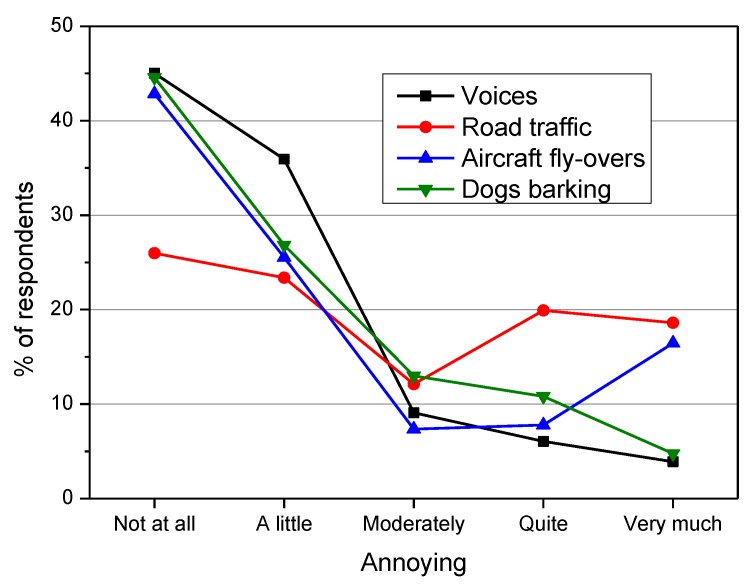
Reported annoyance of sounds heard in the parks.

Voices and dogs barking were the sounds most expected in the park (79% and 63% of respondents. respectively), but noise from aircraft fly-over and road traffic was also expected (33% and 29%, respectively) because of the environmental context within which the parks are located. Nevertheless, these sounds were reported as “highly annoying” by 19% (road traffic) and 16% (aircraft fly-overs) of the respondents, percentages much higher than those observed for dogs barking (5%) and voices (4%) as shown in Figure 7. Thus, as shown in other surveys [4,17], natural sounds (voices and dogs barking in the present study) produce less annoyance than technological sounds (road traffic and aircraft fly-overs in the present study).

The 231 × 9 data matrix formed by the subjective ratings obtained for the nine variables dealing with the quality of vegetation, clean air, cleanliness, security and quietness, and reported annoyance due to voices, road traffic, aircraft fly-overs and dogs barking was subjected to principal component analysis (PCA). The Kaiser-Meyer-Olkin measure of sampling adequacy (MSA test) showed values less than 0.7 for all the variables and for the overall MSA (MSA = 0.58). Thus, the PCA was considered meaningless and not carried out.

**Figure 8:**
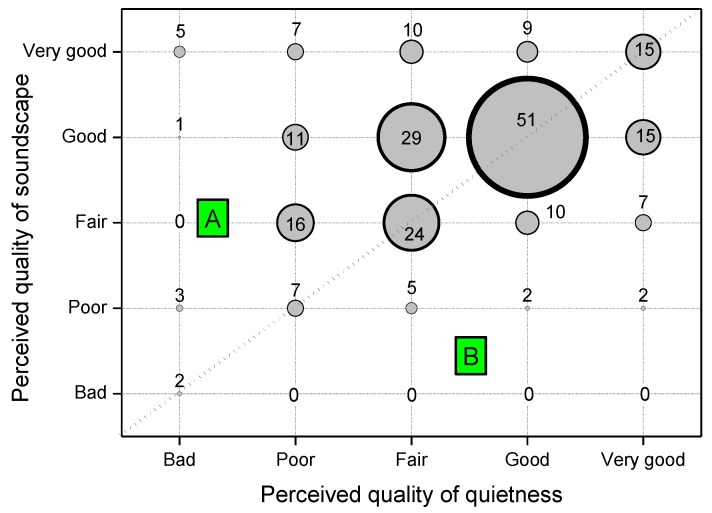
Perceived quality of soundscape *versus* that of quietness.

Figure 8 reports the perceived quality of the soundscape *versus* the quality of quietness in terms of the number of respondents (proportional to the area of circles in Figure 8) for each combination of attributes. The same attribute given for quietness and soundscape (circles along the diagonal) was reported by 99 subjects (43%). Forty-one respondents (18%) judged the quality of quietness better than the soundscape (region B in Figure 8) and 91 (39%) found otherwise (region A in Figure 8). The obtained Spearman’s rank order correlation between quietness and soundscape quality, r_s_ = 0.250, was significant at the 0.01 level.

This result confirms that soundscape has a wider meaning than quietness, the latter being more directly related to either no loud or unwanted sounds. Because sound levels within urban parks are frequently not low, it seems more appropriate to evaluate them from the point of view of “acoustic quality” rather than of “quietness” only [26].

**Figure 9:**
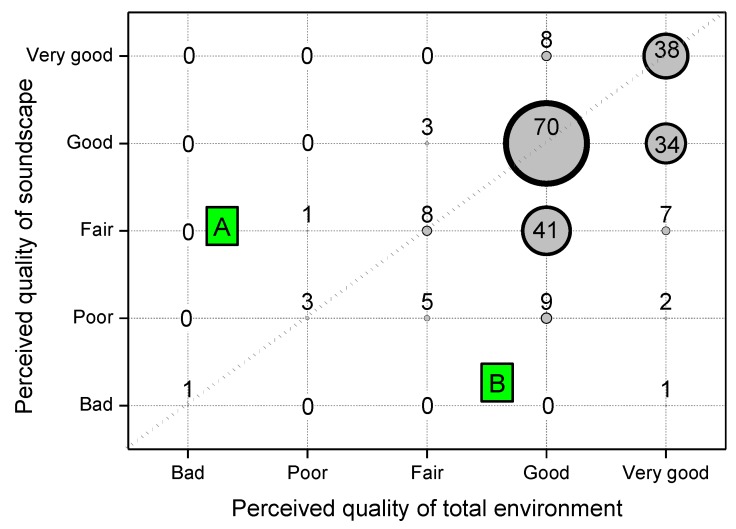
Perceived quality of the soundscape *versus* that of the total environment.

Figure 9 shows the perceived quality of the soundscape *versus* the quality of the total environment in terms of the number of respondents for each combination of attributes (area of circles proportional to this number). The same attribute for environment and soundscape (circles along the diagonal) was reported by 120 subjects (52%), 99 (43%) judged the quality of the total environment better than the soundscape (region B in the plot) and 12 (5%) found otherwise (region A in the plot). The obtained Spearman’s rank order correlation between total environment and soundscape quality, r_s_ = −0.061, was not significant. From this outcome, the perceived quality of the total environment seems to be determined not only by the soundscape but also by other several factors and their interactions concurring to form the sensorial perception.

**Figure 10:**
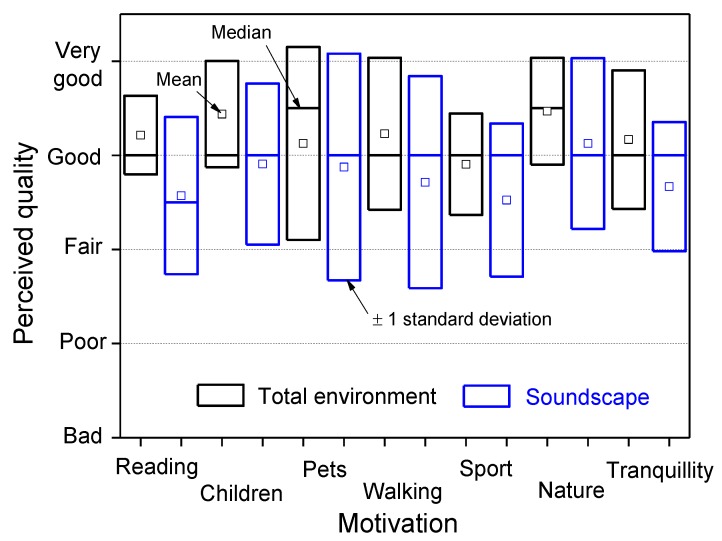
Perceived quality of the soundscape and total environment *versus* motivations for visiting the parks.

Considering the motivation of being in the park, Figure 10 shows that, on average, the perceived quality of the total environment (black boxes with heights corresponding to ±1 standard deviation) is judged better than that of the soundscape (blue boxes) and has lower variability. The median values are around the attribute “good”. Low ratings on the quality of the total environment were observed more frequently for the “sport” motivation, perhaps due to the lack of facilities for such activities, and the corresponding assessment of the perceived soundscape quality was even worse, similar to that given for the “reading” motivation. This result is most likely due to the interference of sounds in the concentration required for reading. Regarding the “tranquillity” motivation, no significant differences (at the 95% confidence level) were observed between ratings on the perceived quality of the total environment and soundscape given on weekdays and Saturdays in the Nord and Sempione parks.

**Figure 11:**
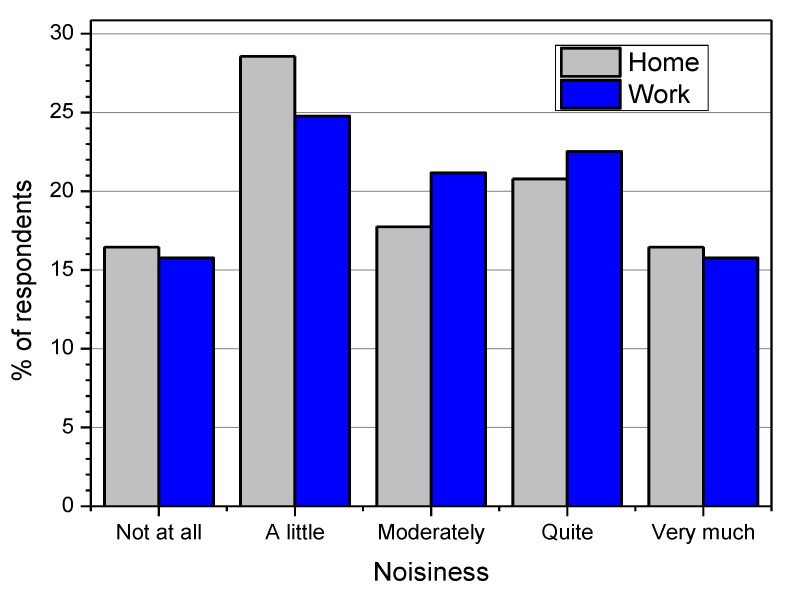
Noisiness of home and workplace reported by the interviewees.

**Figure 12:**
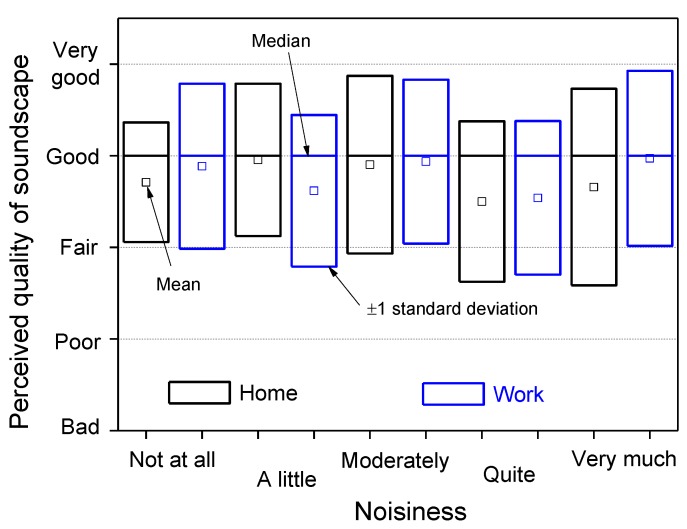
Perceived quality of soundscape *versus* noisiness at home and workplace.

**Figure 13:**
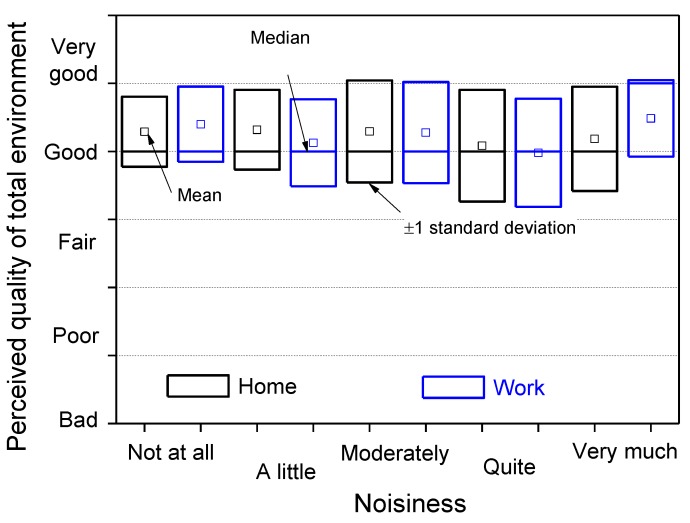
Perceived quality of total environment *versus* noisiness at home and at workplace.

The reported noisiness of the environments where the interviewees live and work is plotted in Figure 11 for each attribute, where the most frequent response was “a little noisy” for both home and workplace. For comparison with the perceived quality of the soundscape, Figure 12 shows the statistics of the responses for every level of noisiness. The median values are independent of the environment (home and work) and correspond to the “good” attribute. The ratings given on the perceived quality of the total environment are more positive, between “good” and “very good” (Figure 13). The median values correspond to the “good” attribute, with the exception of a very noisy workplace, for which the rating on the perceived quality of the total environment is better (median value equals to “very good”).

### 3.3. Relationship between Acoustic Measures and Subjective Responses

**Figure 14:**
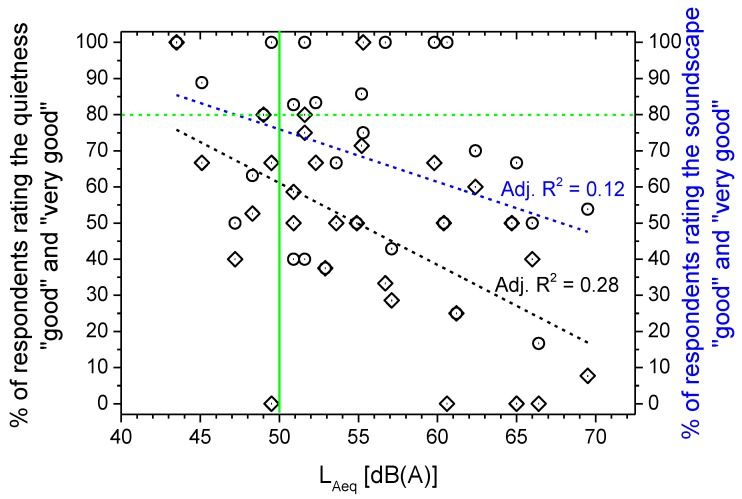
Perceived quality of quietness and soundscape *versus* L_Aeq_ of the parks.

The subjective ratings and the acoustic measurements were compared to reveal potential relationships. The data on “good” and “very good” perceived quality of quietness were pooled, and the corresponding percentages of respondents (black diamonds in Figure 14) are plotted *versus* L_Aeq_. The perceived quality decreases with increasing L_Aeq_, as shown by the linear regression line (dashed black line) at a rate of approximately 7% for every increase of 3 dB in L_Aeq_.

The noise limit of 50 dB(A) for day-time (6–22 h) L_Aeq_ established by the Italian legislation for parks [24] is indicated by the green line in Figure 14. Of the interviewees exposed to L_Aeq_ levels below this limit, 57% rated the perceived quality of quietness in the parks as “good” and “very good”, whereas the percentage falls to 47% for those exposed to higher L_Aeq_ levels.

For each of the 29 sites monitored in the parks, Figure 14 also shows the percentage of subjects reporting a “good” and “very good” perceived quality of the soundscape (blue circles) plotted *versus* the corresponding L_Aeq_. Comparing these data with those corresponding to the perceived quality of quietness (black diamonds) at the same L_Aeq_ level, the soundscape was rated better than quietness in 19 out of 29 sites, worse in three and equal in seven. The good soundscape quality decreases with increasing L_Aeq_, as shown in Figure 14 by the linear regression line (dashed blue line), at a rate of approximately 4% for every increase of 3 dB in L_Aeq_, which is less steep than that observed for quietness. In only 38% of the sites was the percentage of respondents above 80% (dashed green line in Figure 14), the threshold established by the Swedish Environmental Protection Agency for defining a “quiet area” [27], and this percentage decreases to 14% if the Italian day-time limit of L_Aeq_ = 50 dB(A) (green line in Figure 14) is considered.

**Figure 15:**
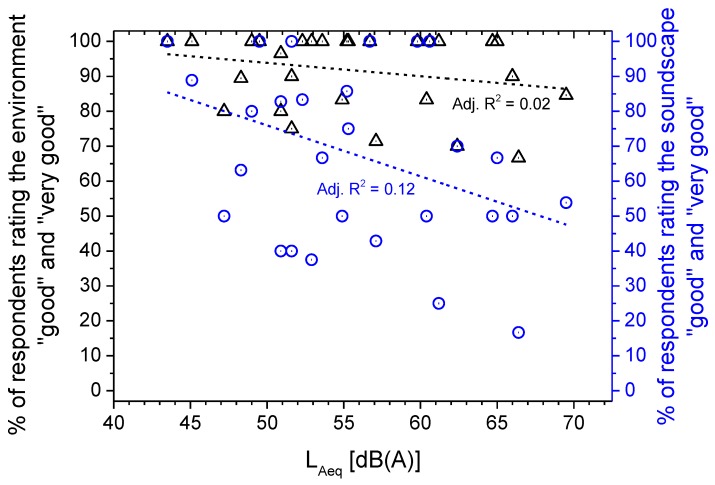
Perceived quality of soundscape and of total environment *versus* L_Aeq_ of the parks.

Figure 15 shows the percentage of subjects reporting “good” and “very good” perceived quality of the total environment (black triangles) plotted *versus* the corresponding L_Aeq_. Comparing these data with those corresponding to the perceived quality of the soundscape (blue circles) at the same L_Aeq_ level, the total environment was rated better than the soundscape in 21 of 29 sites, being worse at one site and equal at seven. The good quality of the total environment decreases with increasing L_Aeq_, as shown in Figure 15 by the linear regression line (dashed black line), at a rate of approximately 1% for every increase of 3 dB in L_Aeq_, which is even less steep than that observed for both soundscape and quietness.

**Figure 16:**
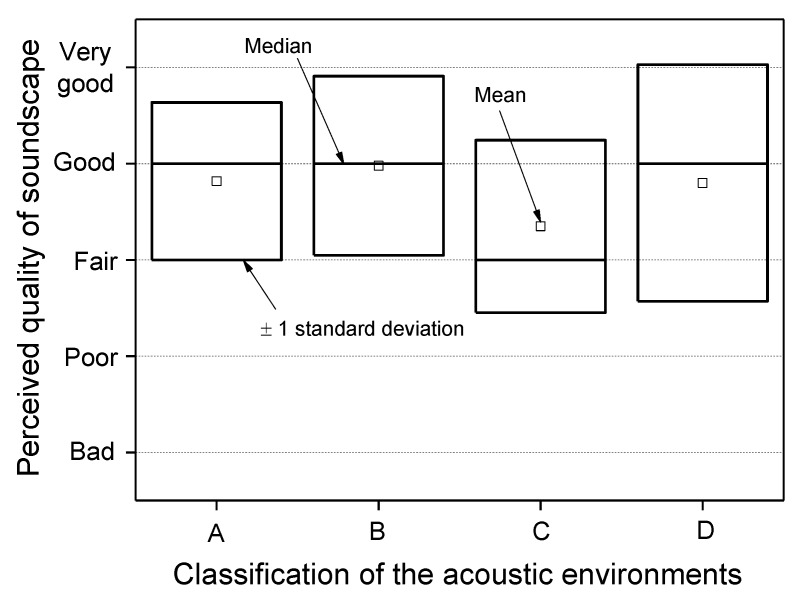
Perceived quality of the soundscape compared with classification based on values of L_A50_ and lg(G).

Further analysis dealt with a comparison of the subjective assessment of the perceived quality of the soundscape with the classification of the acoustic environments described in Figure 4 based on specific values of L_A50_ and lg(G). The results, reported in Figure 16, show that ratings obtained for the sites within region C have a median value, corresponding to “fair” quality, worse than that of the other three regions, which corresponds to “good”. It has to be point out that region C has the greatest boundary value for L_A50_ (61 dB(A)) and values of log(G) below 2.8. However, the subjective ratings do not provide a distinction among the regions as clear as that obtained by the L_A50_ and lg(G) descriptors, as large overlapping occurs when taking into account the rating variability. This reduced discrimination of perceived soundscape quality may be due to the influence on subjective ratings by other non acoustic factors that act as mediators and moderators in the assessment.

**Figure 17:**
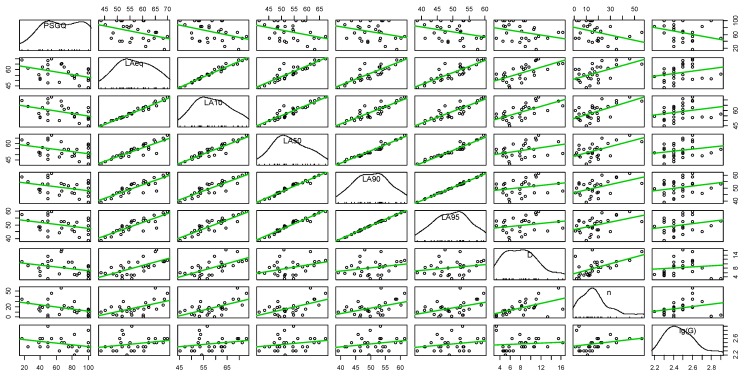
Scatter plot matrix between perceived good soundscape quality (PSGQ), the 7 acoustic descriptors (D = L_A10_ – L_A90_) and number of sound events “n”.

In order to assist future soundscape design in green areas a numerical model to predict the perceived quality would be helpful. For this purpose the correlation matrix of the percentage of respondents reporting a “good” and “very good” perceived quality of the soundscape, the seven sound descriptors already described as input of the cluster analysis and the number of sound events detected by the threshold L_A50_ + 3 dB has been determined. As shown in Table 8 and in Figure 17, there is a strong positive correlation among all the acoustic descriptors, with the exception of lg(G). All the descriptors have a negative correlation with the perceived soundscape good quality (PSGQ), that is their increase leads to a decrease of PSGQ.

**Table 8:** Pearson’s correlation coefficient and their significance (*p*-value).

	PSGQ	L_Aeq_	L_A10_	L_A50_	L_A90_	L_A95_	L_A10_−L_A90_	N. events	lg(G)
PSGQ	1.000								
L_Aeq_	−0.418 (0.012)	1.000							
L_A10_	−0.411 (0.013)	0.984 (0.000)	1.000						
L_A50_	−0.372 (0.023)	0.896 (0.000)	0.933 (0.000)	1.000					
L_A90_	−0.335 (0.038)	0.868 (0.000)	0.892 (0.000)	0.982 (0.000)	1.000				
L_A95_	−0.322 (0.044)	0.859 (0.000)	0.880 (0.000)	0.971 (0.000)	0.999 (0.000)	1.000			
L_A10_ − L_A90_	−0.321 (0.045)	0.660 (0.000)	0.654 (0.000)	0.359 (0.028)	0.241 (0.104)	0.217 (0.129)	1.000		
No. events	−0.396 (0.017)	0.561 (0.001)	0.628 (0.000)	0.582 (0.000)	0.467 (0.005)	0.441 (0.008)	0.567 (0.001)	1.000	
lg(G)	−0.324 (0.043)	0.294 (0.061)	0.279 (0.071)	0.255 (0.091)	0.258 (0.088)	0.263 (0.084)	0.167 (0.193)	0.283 (0.068)	1.000

**Figure 18:**
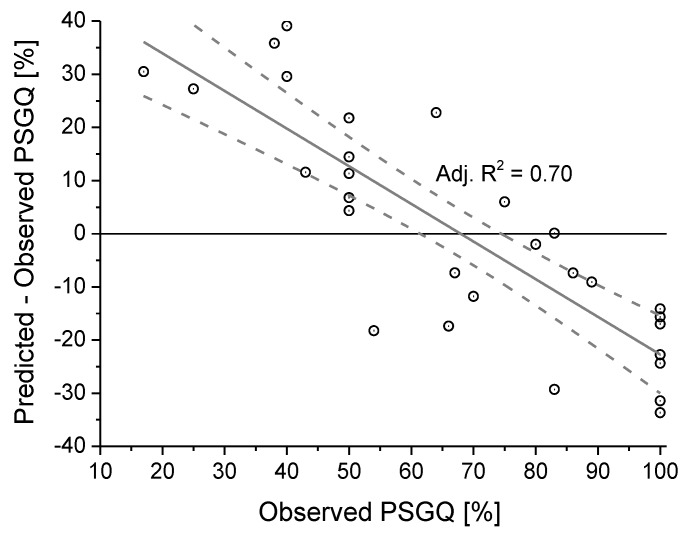
Accuracy of the model obtained by the multiple linear regression.

The model obtained by linear multiple regression, summarized in Table 9, explains only about 30% of the variance. In particular, as shown in Figure 18, the model overstimates the perceived soundscape good quality (PSGQ) at percentage of respondents below 50% (positive differences between predicted and observed PSGQ) and, contrariwise, tends to underestimate at percentages above 50%. The linear regression of the differences between predicted and observed PSGQ is also reported in Figure 18 (grey line), together with bands at 95% confidence level.

**Table 9:** Multiple linear regression analysis relating perceived soundscape good quality (percentage of respondents) and acoustic parameters.

Independent variables	Multiple regression coefficients		Standardized multiple regression coefficients β	R^2^	R^2^ adjusted
	Value *b*	Standard error *b*			
L_Aeq_	−2.915	4.786	−0.826	0.292 *	0.056
L_A50_	5.881	11.618	1.533		
L_A90_	−36.077	45.817	−8.104		
L_A95_	32.951	34.788	7.105		
D = L_A10_ − L_A90_	2.029	6.193	0.272		
No. events n	−0.512	0.640	−0.263		
lg(G)	−32.924	30.655	−0.218		
Intercept	174.845	81.132			

* *p*-value = 0.002 < 0.01.

## 4. Conclusions

The survey carried out in the five urban parks in Milan has shown that the acoustic environment of these areas may reduce, or even lose, the feeling of quietness, in part because of the several sound events observed in the ambient noise produced by numerous sources, from natural to technological. Thus, the important function played by parks in preserving or promoting the health of users can be deteriorated and less efficient. Among the acoustic descriptors considered, the unweighted 1/3-octave spectrum centre of gravity G and L_A50_ performed satisfactorily in discriminating among the acoustic environments, in agreement with the outcome of cluster analysis. Four regions in particular can be distinguished corresponding to intervals for lg(G) and L_A50_ values.

Among the several results obtained from the subjective ratings, the most frequent reason for visiting the park was to seek tranquillity (29%); quietness was the least important aspect for the pleasantness of a park (42%). In the surveyed parks, the quality of the proposed aspects of the park (vegetation, clean air, cleanliness, security and quietness) was mainly judged “good” by the interviewees. Natural sounds (voices and dogs barking) were less annoying than technological sounds (road traffic and aircraft fly-overs).

The quality of quietness was rated worse than that of soundscape by 39% of the respondents, confirming that soundscape has a broader meaning than quietness, the latter being more directly related to no loud or unwanted sounds and being only one aspect of the soundscape. On the other hand, the perceived quality of the total environment was rated better than the soundscape (43% of respondents), likely because the former is determined by several factors (and their interactions) concurrent with determining the sensorial perception.

The collected data on noisiness at home and the workplace show that these environments generally seem to have no influence on the perceived quality of the soundscape, likely because both were mostly rated as little noisy.

The subjective ratings on the perceived quality of the soundscape do not provide a distinction among the acoustic environments as clearly as that obtained by the L_A50_ and lg(G) descriptors, as large overlapping occurs when taking into account the rating variability. This reduced discrimination may be due to the influence on subjective ratings by other non acoustic factors that act as mediators and moderators in the assessment.

The perceived quality of quietness decreases with increasing L_Aeq_ at a rate of approximately 7% for every increase of 3 dB in L_Aeq_. The same trend was observed for that of soundscape and total environment, though less steeply, at a rate of approximately 4% and 1%, respectively, for every 3 dB increase in L_Aeq_.

It has to be addressed that the described survey is only a case study and, therefore, the results are limited to the local situation and they cannot be straightforwardly extended to other environments and green areas. Confirmations and more insights can be obtained from further investigations in order to increase the sampling dimensions. Notwithstanding these limitations, the applied methodology has confirmed that the soundscape approach can provide information useful for planning effective strategies aimed at preserving and improving the quality of urban parks. Because they are even more exposed to pollution and surrounded by extending urban areas, preserving, and hopefully improving, their essential health-promoting function is a challenge but also a need.
